# Health-related quality of life in a nationwide cohort of patients with COPD related to other characteristics

**DOI:** 10.3402/ecrj.v3.31459

**Published:** 2016-05-27

**Authors:** Ingela Henoch, Susann Strang, Claes-Göran Löfdahl, Ann Ekberg-Jansson

**Affiliations:** 1 Angered Local Hospital, Gothenburg, Sweden; 2 Institute of Health and Care Sciences, The Sahlgrenska Academy, University of Gothenburg, Gothenburg, Sweden; 3 Institute of Medicine, The Sahlgrenska Academy, University of Gothenburg, Gothenburg, Sweden

**Keywords:** COPD, quality register, health-related quality of life

## Abstract

**Background:**

In chronic obstructive pulmonary disease (COPD), various factors, such as dyspnoea, obstruction, exacerbations, smoking, exercise capacity, and body mass index, have been found to influence mortality and health-related quality of life (HRQOL). In order to identify subgroups of patients needing special attention, the aim of the present study was to explore the relationships between disease progression factors and HRQOL across COPD stages.

**Methods:**

Baseline registrations from the Swedish COPD register of demographic, clinical, and patient-reported variables of 7,810 patients are presented. Dyspnoea was measured by the modified Medical Research Council (mMRC) dyspnoea scale and HRQOL by the Clinical COPD Questionnaire (CCQ).

**Results:**

This study shows as expected that patients with spirometrically more severe COPD had a significantly higher number of exacerbations and hospitalisations, significantly increasing dyspnoea, significantly decreasing body mass index and exercise capacity, and significantly worsening HRQOL. When adjusting for spirometric stage of COPD, deteriorated HRQOL was predicted by increasing dyspnoea, depression/anxiety, increasing number of exacerbations, and decreased exercise capacity. Further, these data show that an mMRC value of 2 corresponds to a CCQ value of 1.9.

**Conclusion:**

The COPD patients suffered from a significant symptom burden, influencing HRQOL. A surprisingly great proportion of patients in spirometric stages II–IV showed marked changes of CCQ, indicating a need for an improved collaboration between clinical pulmonary medicine and palliative care.

Chronic obstructive pulmonary disease (COPD) is a progressive, restricting, and life-limiting condition which will be the third leading cause of death by the year 2020, in a global perspective, as previously predicted (1). COPD is classified into four spirometric stages according to severity with successively increasing symptom burden from both COPD and comorbidities (2). Although severity of COPD has been associated with deteriorated health-related quality of life (HRQOL) (3), and the spiro metric grouping of severity could be useful for groups of patients, it is not possible to predict the individual patient's experience of living with COPD. However, recent GOLD guidelines recommend combined COPD assessment, where respect is paid to both grading and assessment of symptoms, that is, dyspnoea (4). The assumption is that effective symptom management, as well as exacerbation prophylaxis, will increase patients’ well-being. The individual medication is of course important; inadequate medication increases symptoms, including subjective dyspnoea, whereas good symptomatic treatment reduces the symptoms.

In order to identify patients who need more effective management, two composite measures have been used to predict disease progression; BODE (5), which includes body mass index (BMI), obstruction, dyspnoea, and exercise capacity, measured by 6-min walk test, and DOSE (6), which includes dyspnoea, obstruction, smoking, and exacerbations. BODE (7, 8), DOSE (6), and comorbidity (4) have been found to predict mortality. BODE also predicted hospitalisations (5, 7); BODE (9) and comorbidity (10) have been found to predict HRQOL.

This study attempted to evaluate the various risk factors for deteriorated HRQOL in a Swedish nationwide cohort of registered COPD patients, mainly from primary care centres, but also from specialised out-patient clinics. We also wanted to evaluate the proportion of patients in a real-life situation with moderately or severely impaired HRQOL. We hypothesised that patients with severely impaired HRQOL could be good candidates for improved palliative care; this has not been developed for COPD patients, at least not in our country.

## Methods

Clinical quality registers could be used as a way to examine factors that influence prognosis and HRQOL (11). The Swedish COPD register was introduced in 2009 and the present study comprise data registered until 2012 by the staff at 240 units. Registered items are demographic and clinical variables according to the recommendations of the National Board of Health and Welfare (12). In the present study, the baseline registrations of 7,810 patients in out-patient care are presented, of which 5% of the patients attended specialist pulmonary care, 4% a healthcare centre, and 91% a healthcare centre specialising in COPD. The study was approved by the Ethics Committee at the Department of Medical Ethics in Gothenburg on 7 July 2015 (Dnr: 317-15). As in most registers of this kind, there is a certain number of missing data. However, Clinical COPD Questionnaire (CCQ) values are available for 5,208 patients (67%), of which 5,113 also have modified Medical Research Council (mMRC) values.

### Variables

Demographic variables comprise age, gender, and marital status. Disease-related variables comprise spirometric stage of COPD; obstruction, measured by FEV_1_ as percentage of predicted value; oxygen saturation; number of exacerbations; and number of hospital admissions due to COPD in the past 12 months. Other patient-related variables were comorbidity and BMI. Patient-reported variables were smoking (non-smokers, those who quit smoking, or those still smoking); exercise capacity, measured by number of reported days per week with physical activity; and functional dyspnoea, which was measured by the mMRC dyspnoea scale (13). The mMRC dyspnoea scale is a patient-rated, single-item scale where severity of the dyspnoea experience is reported, ranging from 0, corresponding to ‘not troubled by breathlessness except on strenuous exercise’, to 4, corresponding to ‘breathless when dressing or undressing’. HRQOL was measured by the CCQ (14), a patient-rated questionnaire with 10 items: one item about dyspnoea at rest, one item about dyspnoea during physical activities, two items about how concerned the patient is about the dyspnoea, one item each about cough phlegm, and four items about how the dyspnoea had limited the patient's activities, that is, strenuous physical activities, moderate physical activities, daily activities, and social activities. All items are scored by the patient from 0, ‘never’, to 6, which corresponds to ‘almost all the time’. The mean score of the items is used and the results are interpreted as follows: 4.0–6.0 means a large or very large impact on HRQOL/health status; 2.0–3.9 corresponds to moderate impact on HRQOL, and 0.0–1.9 means no or small impact on HRQOL.

Data about the patients’ medications were also retrieved from the COPD register.

### Data analysis

Descriptive statistics were used for description of the sample and registered treatments, with means and standard deviations (SD) calculated for continuous variables. The categorical variables were presented as numbers and percentages for the total sample (*n*=7,810) and for spirometric stages I, II, III, and IV. Some registrations were missing, resulting in lower numbers of participants in various variables, for example, missing registration data relating to the spirometric stage or for the investigated variable, resulting in a substantially lower number in some of the boxes in Tables 1 and 2. Relationships between different stages of COPD and continuous variables were analysed with Spearman's correlation coefficient, and between stages of COPD and both dichotomous and ordered categorical variables with the Mantel-Haenszel Chi-square test. To investigate the relationships between HRQOL and other variables in the total sample, univariable linear regression analysis with HRQOL, measured by CCQ, as dependent variable and demographic, clinical, and patient-reported measures as independent variables was performed. One analysis of the unadjusted univariable regression is presented along with one adjusted for COPD stage. Significant univariable predictors of HRQOL with *p*<0.001 were entered into two multivariable stepwise forward linear regression analyses with HRQOL measured by the CCQ as dependent variable. One of the models was adjusted for stage of COPD. Due to the large sample and multiple comparisons, only *p*-values of less than 0.001 were considered to be significant. For the adjusted *R*^2^, the proportion of the variance in CCQ that was explained by the predictors was given as a measure of how well the model predicts CCQ.

**Table 1:** Demographic and clinical characteristics of the sample. *N*=7,810 (COPD stage was reported for 7,004 patients)

Demographic variables	Total sample *N*=7,810	Stage I *n*=473 (6.7%)	Stage II *n*=3,408 (48.6%)	Stage III *n*=2,434 (34.8%)	Stage IV *n*=689 (9.8%)	Spearman's Correlation coefficient	Mantel-Haenszel Chi-square test	*p*
Age mean (SD)	69 (9.1)	67.5 (11.3)	68.3 (9.6)	70.1 (8.6)	69.3 (8.4)	0.08		<0.001
Gender, *n* (%)								
Men	3,448 (44.1%)	228 (48.2%)	1,511 (44.3%)	1,067 (43.8%)	322 (46.7%)		0.067	0.796
Women	4,362 (55.9%)	245 (51.8%)	1,897 (55.7%)	1,367 (56.2%)	367 (53.3%)			
Marital status *n*=5,154, *n* (%)								
Living alone	1,934 (38.5%)	81 (31.0%)	746 (34.7%)	696 (41.4%)	211 (44.3%)		61.013	<0.001
Co-habiting	3,083 (61.5%)	181 (69.0%)	1,406 (65.3%)	983 (58.5%)	265 (55.7%)			

Clinical variables	Mean (SD)	Mean (SD)	Mean (SD)	Mean (SD)	Mean (SD)			

FEV% of predicted value	50.3 (20.1)	85.1 (38.6)	59.2 (9.2)	38.5 (6.6)	23.4 (5.2)	−0.88		<0.001
Saturation	95.48 (2.7)	96.7 (1.7)	96.2 (2.1)	95.1 (2.6)	93.7 (3.3)	−0.33		<0.001
Exacerbations during the past 12 months	0.78 (1.4)	0.44 (1.1)	0.55 (1.1)	0.88 (1.4)	1.5 (2.1)	0.22		<0.001
Hospital admissions the past 12 months	0.23 (0.8)	0.06 (0.4)	0.11 (0.6)	0.25 (0.7)	0.59 (1.4)	0.22		<0.001
Body mass index (BMI)	26.0 (5.6)	26.0 (5.2)	26.7 (5.3)	25.6 (5.8)	24.0 (5.7)	−0.15		<0.001

Patient-reported variables	*n* (%)	*n* (%)	*n* (%)	*n* (%)	*n* (%)			

Smoking non-smoker	243 (3.5%)	21 (4.4%)	119 (3.5%)	82 (3.4%)	21 (3.1%)			
Have quit smoking	4,240 (60.7%)	264 (55.9%)	1,955 (57.5%)	1,537 (63.3%)	484 (70.9%)		33.440	<0.001
Still smoking	2,501 (35.8%)	187 (39.6%)	1,325 (39.0%)	811 (33.4%)	178 (26.1%)			

	Mean (SD)	Mean (SD)	Mean (SD)	Mean (SD)	Mean (SD)			

Exercise capacity, days per week	3.3 (2.8)	3.8 (2.7)	3.6 (2.7)	3.2 (2.8)	2.27 (2.6)	−0.12		<0.001
Functional dyspnoea (MRC)	1.88 (1.2)	1.21 (1.0)	1.48 (1.1)	2.18 (1.2)	2.97 (1.1)	0.39		<0.001
Quality of life according to CCQ	1.8 (1.2)	1.4 (1.1)	1.5 (1.0)	2.05 (1.2)	2.7 (1.2)	0.33		<0.001

Spearman's correlation coefficient is presented between continuous variables and stage.

**Table 2:** Univariable linear regression analysis with quality of life measured by CCQ as dependent variable and demographic and clinical characteristics, comorbidity, and patient-reported variables as independent variables, unadjusted and adjusted for stage of COPD

	Total sample (n=7,810)			
	
	Unadjusted		Adjusted for stage of COPD	
		
	β (95% CI)	*p*	β (95% CI)	*p*
Demographics				
Age (10 years interval)	0.059 (0.025, 0.094)	0.001	0.030 (−0.003, 0.063)	0.074
Gender (1=men, 2=women)	0.009 (−0.055, 0.073)	0.783	0.004 (−0.057, 0.065)	0.885
Marital status (1=living alone, 2=co-habiting)	−0.159 (−0.219, −0.099)	<0.001	−0.092 (−0.149, 0.035)	0.002
Clinical variables				
FEV_1_% of predicted value	−0.015 (−0.017, −0.014)	<0.001	−0.004 (−0.006, 0.002)	<0.001
Exacerbations during the past 12 months	0.241 (0.220, 0.263)	<0.001	0.189 (0.167, 0.210)	<0.001
Hospital admissions the past 12 months	0.309 (0.269, 0.348)	<0.001	0.224 (0.183, 0.264)	<0.001
Body mass index (BMI)	0.001 (−0.005, 0.007)	0.709	0.012 (0.006, 0.018)	<0.001
Comorbidity				
Heart failure	0.591 (0.485, 0.696)	<0.001	0.503 (0.401, 0.605)	<0.001
Ischemic heart disease	0.276 (0.188, 0.363)	<0.001	0.251 (0.167, 0.334)	<0.001
Stroke	0.171 (0.025, 0.316)	0.022	0.122 (−0.021, 0.264)	0.094
Hypertension	0.027 (−0.038, 0.093)	0.417	0.006 (−0.057, 0.068)	0.854
Atrial fibrillation	0.277 (0.159, 0.396)	<0.001	0.278 (0.164, 0.392)	<0.001
Diabetes	0.137 (0.037, 0.236)	0.007	0.155 (0.060, 0.249)	0.001
Osteoporosis	0.410 (0.305, 0.515)	<0.001	0.250 (0.148, 0.352)	<0.001
Depression/anxiety	0.558 (0.472, 0.643)	<0.001	0.552 (0.440, 0.604)	<0.001
Lung cancer	0.185 (−0.166, 0.536)	0.301	0.025 (−0.312, 0.361)	0.886
Alfa-1-antitrypsin deficiency	0.426 (0.047, 0.804)	0.028	0.348 (−0.024, 0.719)	0.066
Patient-reported variables				
Smoking, 0=non-smoker, 1=have quit smoking, 2=still smoking	−0.014 (−0.075, 0.046)	0.641	0.055 (−0.002, 0.113)	0.060
Exercise capacity, days per week	−0.119 (−0.130, −0.107)	<0.001	−0.101 (−0.112, −0.090)	<0.001
Functional dyspnoea (MRC)	0.628 (0.609, 0.647)	<0.001	0.558 (0.567, 0.610)	<0.001

## Results

**Fig. 1:**
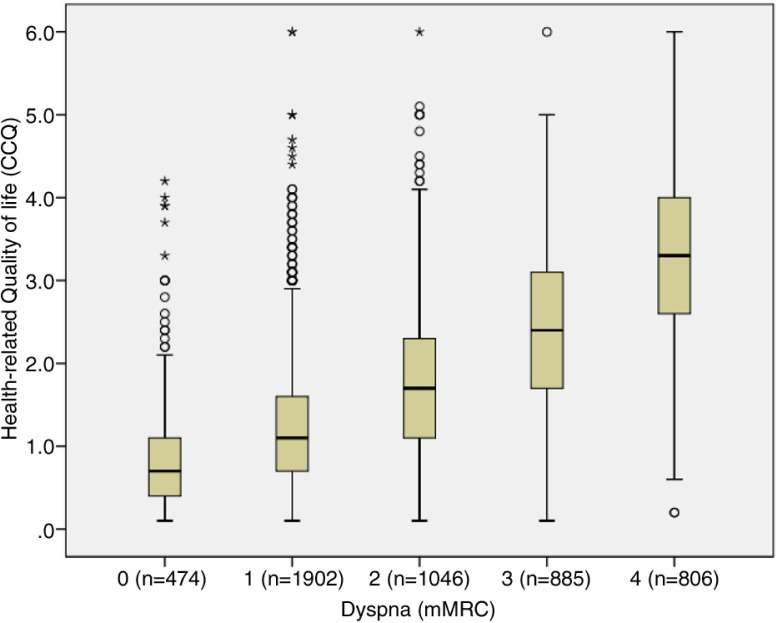
Box plot showing the distribution of CCQ related to grades of mMRC.

**Fig. 2:**
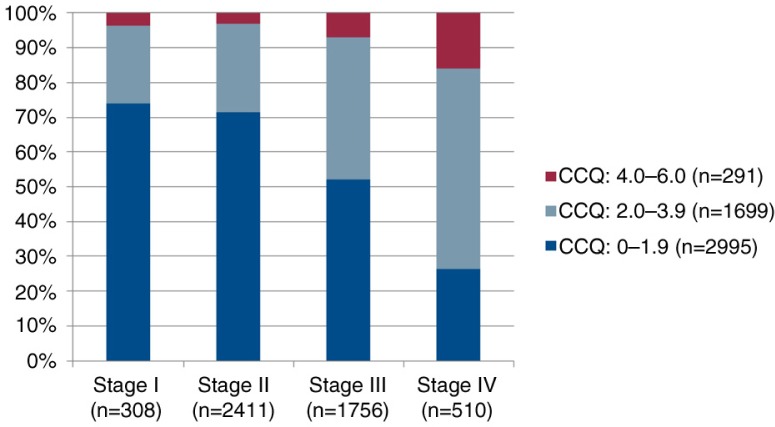
Distribution of CCQ levels related to spirometric stages. 4.0–6.0 means large or very large impact on HRQOL/health status; 2.0–3.9 corresponds to moderate impact on HRQOL, and 0.0–1.9, no or small impact on HRQOL.

Demographic and clinical characteristics of the patients are presented in Table 1. A majority of the patients had COPD stage II (49%), 57% were diagnosed within the past 5 years, and 56% were women. Median age for the total sample was 70 years, with a range of 30–98. The median subjectively rated dyspnoea degree, according to the mMRC, was 2, corresponding to ‘becoming breathless when walking on flat ground at the same speed as a person of the same age’. The median HRQOL, according to the CCQ, was 1.6, which corresponds to ‘little or no impact on HRQOL’. When divided into stages, the patients with stage I reported median of 1.1; patients with stage II, median of 1.3; patients with stage III, median of 1.9; and patients with stage IV, median of 2.8, corresponding to ‘moderate influence on HRQOL’. Figure 1 shows that the variation of CCQ to dyspnoea is wide and Fig. 2 shows that the proportion of moderately or severely impaired HRQOL is high, but surprisingly, not only in stages III and IV. We also wanted to evaluate the correspondence between dyspnoea (mMRC) and HRQOL as measured by CCQ. We anticipated a linear relationship and found that an equation of CCQ=0.63+0.63*mMRC, by which 2 in mMRC corresponds to CCQ 1.9 (with an extremely low 95% CI).

**Fig. 3:**
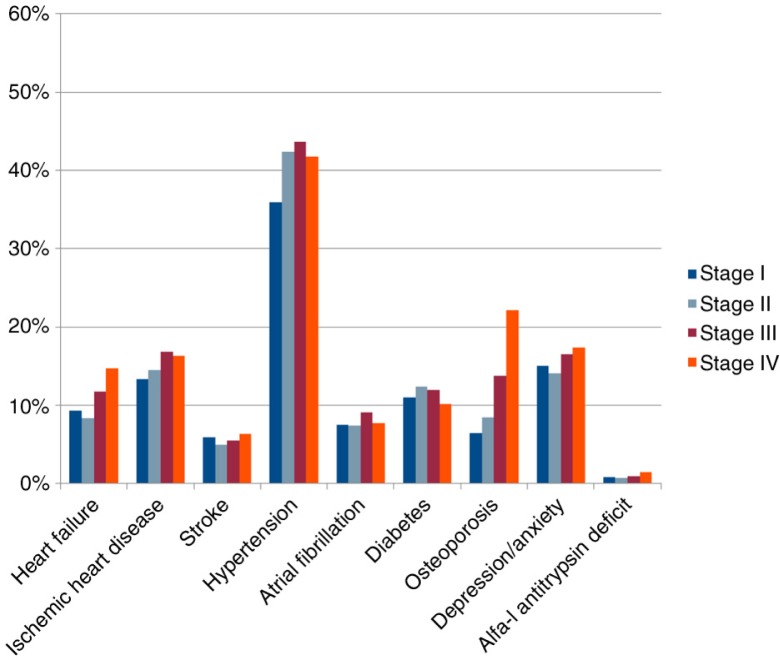
Comorbidities across stages.

The median number of reported days per week with physical activity was 3, varying between 0 and 7. There were significant differences across stages in that those patients with more severe disease had much less physical capacity, as expected (Table 1). In relation to comorbidity, there were significant differences in prevalence across stages in heart failure and osteoporosis, in that they were more prevalent in more severe disease (Fig. 3). Depression/anxiety was more prevalent in stage I than in stage II, but even more prevalent in stages III and IV.

When analysing relationships between subjectively measured dyspnoea with the mMRC and spirometric measurements (FEV_1_ as % of predicted value), a significant correlation of −0.41 (*p*<0.001) was found.

**Fig. 4:**
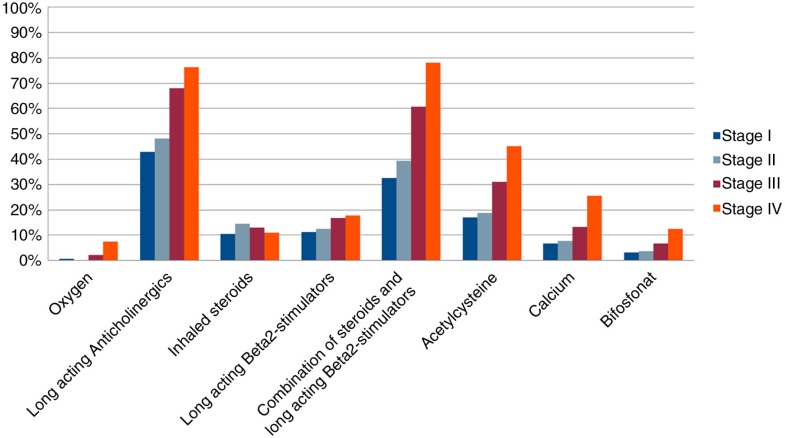
Distribution of medication across COPD stages.

As shown in Fig. 4, patients in later stages were prescribed more extensive medical treatment.

There were univariable relationships between HRQOL and marital status, FEV_1_ as % of predicted value, exacerbations and hospitalisations due to COPD, most comorbidities, physical activity, and functional dyspnoea, although some of the beta coefficients were low (Table 2). When adjusting for stage of COPD, predictors of HRQOL were FEV_1_ as % of predicted value, exacerbations and hospitalisations due to COPD, BMI, most comorbidities, physical activity, and functional dyspnoea.

Significant variables from the linear univariable regression analyses adjusted for spirometric stage of COPDwere entered in linear multivariable stepwise regression analyses, first unadjusted and then adjusted for stage of COPD (Table 3). In the first model without COPD, FEV_1_ as % of predicted value, exacerbations, depression/anxiety, exercise capacity, and functional dyspnoea were independent predictors to HRQOL. When adjusting for spirometric COPD stage, the multivariable model, of course, lost FEV_1_ as a predictor for HRQOL but all the other factors remained. In this model, dyspnoea was the strongest factor for deterioration, much more important than, for example, the history of exacerbations.

**Table 3:** Multivariable stepwise linear regression analysis with quality of life measured by the CCQ as dependent variable and variables which became significant at *p*<0.001 in the univariable analyses as independent variables

		Total sample (*n*=7,810)			
		
	Independent variables	Unadjusted		Adjusted for stage of COPD	
	
Adjusted *R*^2^		0.477		0.480	

		Unadjusted β (95% CI)	*p*	Adjusted β (95% CI)	*p*
Clinical variables	FEV% of predicted value	−0.002 (−0.004, −0.001)	0.001		NS
	Exacerbations during the past 12 months	0.128 (0.105, 0.151)	<0.001	0.125 (0.102, 0.148)	<0.001
Comorbidity	Depression/anxiety	0.202 (0.113, 0.291)	<0.001	0.207 (0.118, 0.295)	<0.001
Patient-reported variables	Exercise capacity, days per week	−0.039 (−0.051, −0.027)	<0.001	−0.039 (−0.051, −0.027)	<0.001
	Functional dyspnoea (MRC)	0.541 (0.512, 0.570)	<0.001	0.530 (0.500, 0.560)	<0.001

## Discussion

This study shows in a real-life setting that there is a widespread level of impairment of HRQOL in patients with COPD, regardless of their spirometric stage. This has earlier been shown, and it emphasises the importance of individual evaluation of patients when treating their disease, taking into account dyspnoea and comorbidities such as depression/anxiety, exacerbations, and exercise capacity. This is, to some extent, in agreement with the current GOLD guidelines for the treatment of COPD. This study also shows that a significant portion of patients experience a more severely impaired HRQOL to some extent over different spirometric stages.

The importance of dyspnoea to HRQOL is in agreement with earlier studies (15), and it is one possible way to evaluate patients in the GOLD matrix (7). However, the current GOLD classification defines dyspnoea as measured by mMRC along with evaluated questionnaires such as the CAT (COPD assessment test) and CCQ. The levels chosen for equal symptomatic burden have not been well documented, and recently, a paper publishing study results from Barcelona showed that, for the CAT, the value of 17 (out of a 40 possible points) corresponded to mMRC 2 with regard to mortality (16). Our data suggest that for the CCQ, the corresponding value is 1.9 (of a possible 6). The conclusion therefore would be that the suggested ways to evaluate symptomatic burden according to the GOLD guidelines do not show a good correspondence.

In previous studies, BODE (8), DOSE (6), and comorbidity (4) were found to predict disease progression. Dyspnoea, exercise, and exacerbations as predictors coincide with the results from the present study. In BODE, DOSE, and in the present study, dyspnoea was an important predictor of HRQOL. On a total cohort level, our data show the great impact of dyspnoea on HRQOL, and may be, somewhat surprisingly, a much stronger factor than exacerbations. Both of these factors are well represented in the CCQ questionnaire and would probably show up in a similar manner. The low impact (although statistically significant) of physical capacity in our data is probably due to a close correlation between functional dyspnoea and exercise capacity. A strong factor to influence HRQOL is the occurrence of depression/anxiety, as has also been shown in other studies (17, 18), as well as in the present study, where HRQOL was predicted by depression in patients with COPD. This relationship, in a meta-analysis, seems to correspond more strongly with the St. George's Respiratory Questionnaire (SGRQ) than in our study of CCQ (17). Using yet another questionnaire, the Chronic Respiratory Questionnaire (CRQ), in a primary care setting, the impact of depression was also seen, however, at the same level that we see in our data (18).

Hospital admissions were a predictor of HRQOL, when unadjusted for stage of COPD. It is well-known that the number of exacerbations treated in hospital increases with any severity grading. The number of exacerbations predicts HRQOL, which probably indicates that there is a potential for further treatment to prevent exacerbations. Prevention of exacerbations prevents deterioration of health status (19) and a patient-centred care approach could be used to avoid hospital admissions (20).

Physical activity decreased to only a mean of 2.7 days with countable exercise per week in stage IV, and for some patients, physical activity might not even be possible. In the other stages, the mean value for exercise is 3.8–3.2 days per week with a 30-min walk. This result emphasises the need for early onset of pulmonary rehabilitation with prevention of deteriorated physical activity and falling HRQOL. This also improves dyspnoea, fatigue, exercise capacity, and HRQOL in the early stage (21).

The importance of the psychological status of the patients, as indicated by the figures for the impact of depression/anxiety, is certainly also due to the increasing limitations to everyday life for these patients. In a previous interview study, patients with COPD in late stages were found to experience both death anxiety, when living with a life-limiting illness with severe symptoms, and also anxiety related to be forced to live a life with dyspnoea and severe limitations (22). Patients’ experiences of anxiety and depression need to be much better acknowledged by healthcare professionals.

This study shows that patients with COPD suffer from a high burden of symptoms, especially breathlessness. As expected, the breathlessness increased with deteriorating disease. The importance of patient-reported dyspnoea, decreasing physical capacity, increasing number of exacerbations, and higher prevalence of depression indicate a need for more comprehensive care, including physical, psychological, social, and existential domains (23). In stage IV, almost 20% of the patients suffer from a severely impaired HRQOL, and also in stage II, the corresponding proportion is around 8%. A palliative approach with four dimensions (physical, psychological, social, and spiritual) strives to see the person as a whole and it is known that, among other things, psychological and existential issues are common – and these are questions that palliative care has been used to dealing with (24). A possibility to alleviate the heavy symptom burden, especially in late stages, would be to cooperate with the palliative care service. The common occurrence of comorbidities, as also seen in this study, further emphasises the need for palliative approaches to these patients. Although the symptom burden for COPD patients could be comparable and even worse than it is for patients affected by malignant diseases (15, 25), they are seldom candidates for palliative treatment (25, 26). We therefore argue for a close collaboration between clinical pulmonology and palliative care centres.

As COPD is found to be underdiagnosed (27), and only 20–30% of patients fulfil the criteria for COPD as correctly identified, the amount of suffering in this patient group could be expected to be even higher. When compared with the study by Lindberg et al. (27), the present sample had more advanced disease and the register, to a larger extent, had identified the patients who were affected by more severe disease. However, the National Board of Health and Welfare (12) have stated that it is important to identify patients earlier in order to prescribe appropriate treatment and interventions earlier. Only 7% of the present sample was diagnosed with mild COPD, which is associated with the possibility to delay disease deterioration.

### Limitations

There are several limitations to a register study such as ours. The main limitation is that it covers centres in our country that started to register their patients relatively early. However, we do have a representative number of patients with different severities. A register can obviously not include undiagnosed cases, and therefore it does not show the whole national burden of the disease.

The comprehensive registered variables are, on the one hand, a strength for the register, but could, on the other hand, be an obstacle for healthcare professionals to start the registration of a patient in a slim-lined organisation.

Relating to a specific questionnaire to measure HRQOL such as the CCQ in this report could bias the results as the specific questionnaire more or less emphasises specific aspects of the disease. However, other studies have shown a good correlation for the CCQ with other questionnaires, such as the SGRQ (28).

## Conclusions

The registered patients affected by COPD suffered from a symptom burden with increasing dyspnoea, depression, and anxiety; function deficit; and increasing number of exacerbations, strongly influencing their HRQOL. The symptom burden and the exacerbations history seem to be linear events with close correlation. The spread of HRQOL within the same spirometric stages is also wide, and an individual evaluation is of uttermost importance when suggesting treatment. Within the more severe groups there is a considerable proportion of patients with a heavy symptomatic burden, suggesting that early collaboration between rehabilitation and palliation units would be beneficial for selected patients.
